# The impact of death literacy on self-management behaviors in gastric cancer patients: the chain mediating role of psychological adaptation and cognitive reappraisal

**DOI:** 10.3389/fpubh.2025.1735804

**Published:** 2026-01-05

**Authors:** Minyi Shi, Chengxi Xia, Yuxin Ye

**Affiliations:** 1 Department of Gastroenterology, Taizhou First People's Hospital, Taizhou, China; 2 Huangyan Hospital, Wenzhou Medical University, Taizhou, China; 3 Tongde Hospital of Zhejiang Province Affiliated to Zhejiang Chinese Medical University (College of Integrated Traditional Chinese and Western Medicine Clinical Medicine), Hangzhou, China

**Keywords:** gastric cancer patients, death literacy, self-management behaviors, psychological adaptation, cognitive reappraisal

## Abstract

**Background:**

Death literacy—defined as the knowledge, skills, and attitudes that enable individuals to understand, communicate, and act effectively on issues related to death, dying, and bereavement—plays an important role in cancer patients' psychological regulation and coping behaviors. However, empirical evidence linking death literacy to self-management behaviors in gastric cancer patients remains limited.

**Methods:**

This study employed a cross-sectional design and used convenience sampling to recruit 501 gastric cancer patients from three tertiary Grade A hospitals in Zhejiang Province between August and September 2025. All participants completed the Death Literacy Scale, Self-Management Scale, Psychological Adaptation Scale, and Cognitive Reappraisal Scale. Process Model 6 was used to analyze the chain mediating effects of psychological adaptation and cognitive reappraisal.

**Results:**

Death literacy was positively correlated with self-management behaviors (*r* = 0.495, *p* < 0.001). Bootstrapping analysis confirmed significant indirect effects via psychological adaptation [β = 0.109, SE = 0.037, 95% CI = (0.036, 0.181)] and cognitive reappraisal [β = 0.106, SE = 0.029, 95% CI = (0.054, 0.170)], as well as a significant sequential mediation pathway [β = 0.068, SE = 0.018, 95% CI = (0.031, 0.102)], jointly explaining 59.75% of the total effect. The results suggest that higher death literacy enhances psychological adaptation and facilitates positive emotion regulation, thereby promoting active and sustained self-management behaviors.

**Conclusions:**

Death literacy functions as a positive psychological resource contributing to adaptive coping and health management among gastric cancer patients. Strengthening patients' understanding and acceptance of death may improve psychological adaptation and cognitive reappraisal, ultimately fostering better self-management and quality of life.

## 1 Introduction

Gastric cancer is a malignant tumor originating from the epithelial cells of the gastric mucosa (1, 2), and it remains one of the primary malignant tumors of the digestive system worldwide, exhibiting persistently high incidence and mortality rates (3, 4). Postoperative nutritional disorders, pain, and functional impairments further exacerbate the disease burden (5, 6). These factors intensify psychological distress in gastric cancer patients, such as anxiety, depression, and post-traumatic stress disorder, which not only diminish patients' quality of life but also weaken treatment adherence and overall prognosis (7, 8). Previous research has found that cancer patients' perceptions of death and their coping strategies play a positive role in alleviating psychological distress, particularly death literacy (9–11). Death literacy refers to the knowledge, skills, and attitudes individuals possess when acquiring, understanding, and applying information related to death, dying, and bereavement (12, 13). For cancer patients, those with high death literacy are often better able to rationally confront disease threats, thereby promoting positive psychological adaptation and behavioral adjustments (14). However, existing psychological interventions for gastric cancer primarily focus on symptom management (15), overlooking the potential value of death literacy in psychological regulation and behavioral responses. Therefore, this study analyzes how death literacy in gastric cancer patients influences their behavioral responses, which holds significant clinical implications.

Death literacy aims to help individuals build cognitive and action capabilities in facing death (16). Patients with high death literacy typically exhibit greater psychological resilience, better integration of medical information, and active participation in decision-making (17, 18). For instance, death literacy in gastric cancer patients often reduces death anxiety and improves life satisfaction through rational cognition of the disease reality (19). This is primarily because death literacy serves as a protective resource, helping patients mitigate the psychological impact of the disease and maintain psychological stability (20). Although previous studies have proposed interventions such as psychological education, medical planning, and death dialogues to improve patients‘ psychological adaptation and treatment engagement (21, 22), few have analyzed the relationship between patients' death literacy and self-management behaviors, especially in gastric cancer patients.

Self-management behaviors refer to a series of interactive processes in which patients actively participate in disease management, including medication adherence, dietary adjustments, symptom monitoring, physical exercise, and regular follow-ups (23–25). In gastric cancer patients, effective self-management behaviors are closely associated with improved survival rates, reduced complications, and enhanced quality of life (26). For example, strict dietary management and symptom recording can help alleviate treatment side effects (27), while positive psychological adjustments can enhance treatment motivation (28). However, gastric cancer patients often face barriers to self-management due to disease progression and treatment side effects, such as fatigue, pain, and emotional distress, which reduce treatment adherence (29). According to social cognitive theory, self-management behaviors are influenced by various factors, including individual cognition, emotional states, and social support (30). Psychological factors such as death literacy may indirectly enhance self-management behaviors by affecting patients' cognitive evaluations and emotional responses (31). Therefore, we posit that patients with high death literacy may improve their self-management behaviors through treatment planning and clear disease cognition. Additionally, self-management behaviors in gastric cancer patients often decline significantly in the advanced stages of the disease, which is correlated with death anxiety and adaptation difficulties. Based on the above theoretical analysis, this study will conduct a cross-sectional investigation to further explore: (1) the relationship between death literacy and self-management behaviors in gastric cancer patients; (2) the internal mechanisms between death literacy and self-management.

Psychological adaptation is the process by which individuals achieve psychological balance through cognitive and emotional adjustments when facing major life stressors (32, 33). In gastric cancer patients, psychological adaptation often manifests as disease acceptance, meaning-making, and disease regulation, which are closely related to patients' mental health and functional recovery (34, 35). Gastric cancer patients commonly experience prolonged psychological fluctuations due to postoperative changes in digestive function and recurrence risks (36). For example, Christodoulidis et al. (37) found that ~57% of gastric cancer patients fail to adapt effectively within 1 year after diagnosis, leading to increased depression rates. Therefore, improving patients' psychological adaptation is crucial for enhancing quality of life. Death literacy accelerates the adaptive process by providing death-related knowledge and reducing uncertainty fears (38). Specifically, patients with higher death literacy, while recognizing the finitude of life, reevaluate life values and goals, fostering positive life attitudes and promoting emotional integration and psychological acceptance. Patients with good psychological adaptation, after emotional adjustment and cognitive integration, can rebuild self-efficacy and a sense of control, thereby participating more proactively in self-management (39). According to the Health Belief Model, gastric cancer patients with higher levels of psychological adaptation typically believe more strongly that their behaviors can influence health outcomes, exhibiting greater persistence and adherence in dietary control, follow-up management, and psychological rehabilitation training (40).

Cognitive reappraisal is an emotion regulation strategy that involves individuals adjusting their emotional experiences and behavioral responses by altering their cognitive interpretations of situations or events, emphasizing the cognitive reconstruction of event meanings (41, 42). In gastric cancer patients, disease diagnosis and treatment can evoke strong negative psychological states (43), but cognitive reappraisal allows patients to redefine the meaning of the disease—such as viewing treatment as a recovery process or understanding disease experiences as opportunities for life growth—thereby transforming negative emotions into positive psychological experiences (44). However, gastric cancer patients with higher death literacy are better able to confront the finitude of life, viewing death as a natural process rather than an uncontrollable threat (45), thus reducing negative biases in emotional responses and making them more likely to adopt positive cognitive reappraisal strategies. According to cognitive appraisal theory, individuals undergo primary and secondary appraisals when coping with stressful events; patients with high death literacy experience weaker threat perceptions in the primary appraisal stage and are more inclined to believe they possess coping resources and control in the secondary appraisal stage (46). When cognitive reappraisal optimizes emotional states, patients can maintain rational and calm attitudes in facing disease fluctuations or treatment side effects, continuing to execute health plans and avoiding behavioral interruptions due to emotional exhaustion (47). Therefore, this study posits that death literacy promotes the use of cognitive reappraisal strategies by altering individuals' meaning assessments and emotional understandings of death; in turn, cognitive reappraisal drives the formation of higher-quality self-management behaviors through positive emotion generation and intrinsic motivation stimulation.

Based on the above theoretical analysis, this study further proposes that death literacy may influence self-management behaviors in gastric cancer patients through a chain mediating pathway involving psychological adaptation and cognitive reappraisal. According to Lazarus & Folkman's stress-coping theory, individuals undergo a continuous psychological processing sequence from cognitive appraisal to emotional response to coping behavior under disease threats (48, 49). High death literacy enables patients to approach death-related issues with greater rationality and control, thereby reducing anxiety from primary threat appraisals, promoting internal emotional integration and psychological balance, which manifests as higher levels of psychological adaptation. Elevated psychological adaptation provides patients with a more stable emotional foundation and greater cognitive flexibility, allowing them to employ cognitive reappraisal strategies in the secondary appraisal stage to redefine the meanings of disease and death, transforming negative experiences into opportunities for growth and action. Cognitive reappraisal, while maintaining positive emotions, enhances intrinsic motivation and self-efficacy for health management, thereby promoting sustained and autonomous self-management behaviors. Thus, psychological adaptation and cognitive reappraisal collectively form a continuous psychological pathway through which death literacy influences self-management behaviors. Psychological adaptation provides psychological resources for cognitive reappraisal through emotional integration, while cognitive reappraisal further converts emotion regulation into actual health behaviors, forming a “death literacy → psychological adaptation → cognitive reappraisal → self-management behaviors” chain mediating mechanism. This pathway reveals the psychological transformation process from cognitive awareness to behavioral execution, providing theoretical support for understanding psychological regulation patterns and intervention strategies in gastric cancer patients.

Based on the above analysis, this study proposes the following hypotheses:

**H1:** Death literacy in gastric cancer patients has a significant positive predictive effect on self-management behaviors;
**H2:** Psychological adaptation has a significant partial mediating effect on the relationship between death literacy and self-management behaviors in gastric cancer patients;
**H3:** Cognitive reappraisal has a significant partial mediating effect on the relationship between death literacy and self-management behaviors in gastric cancer patients;
**H4:** Psychological adaptation and cognitive reappraisal have a significant chain mediating effect on the relationship between death literacy and self-management behaviors in gastric cancer patients.

## 2 Methods

To verify the aforementioned hypotheses, this study adopted a cross-sectional design to explore the relationships between death literacy and self-management behaviors in gastric cancer patients, and to validate the chain mediating roles of psychological adaptation and cognitive reappraisal. The study constructed a psychological mechanism model based on stress-coping theory, with data collected through questionnaire surveys. Convenience sampling was employed, and data collection occurred from August 1 to September 28, 2025.

### 2.1 Participants

#### 2.1.1 Ethical considerations

This study was approved by the Academic Ethics Committee of Taizhou First People's Hospital. Throughout the research process, participants' privacy rights and data confidentiality were strictly protected. Prior to completing the questionnaires, participants received written and oral explanations, clearly outlining the study purpose, participation rights, and freedom to withdraw. The study adhered to the ethical principles of the Declaration of Helsinki, and all participants voluntarily participated and signed written informed consent forms.

#### 2.1.2 Data recruitment process

The study sample was drawn from gastric cancer patients at three tertiary Grade A hospitals. Researchers collaborated with oncology surgery and endoscopy centers, where research nurses screened eligible patients based on medical records and conducted initial communications. Eligible patients who expressed willingness to participate completed the questionnaires during inpatient stays or follow-up visits. The data collection process included three stages: (1) Researchers informed patients of the study purpose in outpatient clinics or wards and assessed their condition; (2) Willing participants signed informed consent forms and completed the questionnaires; (3) Researchers checked the completeness of the questionnaires on-site and collected them. To minimize selection bias, data collection encompassed patients at different disease stages (Stages I–IV) and treatment modalities (surgery, chemotherapy, and combined treatments).

#### 2.1.3 Minimum sample size

This study used *G* ^*^ Power 3.1.9.7 software to calculate the sample size for validating the mediation model involving death literacy, psychological adaptation, cognitive reappraisal, and self-management behaviors. Based on experiences from similar psychological studies (medium effect size, *f*^2^ = 0.15), the test was set as linear multiple regression analysis (multiple predictors model), with significance level α = 0.05, power (1–β) = 0.95, and 13 independent variables (including control variables), yielding a minimum required sample size of *N* = 119. Considering that the study model involved chain mediation analysis and structural equation modeling, which demand larger samples for stability, the sample size was adjusted upward by approximately three times based on model complexity and the number of latent variables to ensure model stability and reliable parameter estimation. Thus, the final minimum sample size was determined to be 357 participants.

#### 2.1.4 Inclusion and exclusion criteria

Inclusion criteria: (1) Confirmed diagnosis of primary gastric cancer (Stages I–IV) via gastroscopic biopsy and pathology; (2) Age ≥ 18 years, with clear consciousness, language communication ability, and basic literacy to independently understand and complete questionnaires; (3) Stable medical condition, not in acute complications or critical states; (4) Currently undergoing or having completed surgery, chemotherapy, or combined treatment for at least 1 month to enhance patients' basic cognition and psychological response experiences; (5) Voluntary participation in the study and signed written informed consent.

Exclusion criteria: (1) Patients with concurrent other malignant tumors or severe organic diseases (e.g., cirrhosis, severe heart failure); (2) Presence of severe cognitive impairment, language expression disorders, or history of mental illness affecting comprehension and expression; (3) Currently receiving psychological therapy or in intensive care units; (4) Questionnaires showing strong response consistency.

#### 2.1.5 Study sample

A total of 545 gastric cancer patients were recruited, of which 24 had concurrent severe organic diseases, including severe coronary heart disease, cirrhosis, and severe bronchial diseases; 3 participants had severe language expression disorders, such as slurred speech. During data cleaning, 17 questionnaires with strong response consistency were excluded. Thus, the effective sample size for this study was 501 gastric cancer patients, with an effective rate of 91.93%. Among them, there were 299 male patients (57.30%) and 202 female patients (38.70%). A total of 284 patients had junior high to high school education (54.40%), 196 patients had income between 3,001–5,000 yuan (37.50%), and 262 patients resided in urban areas (50.20%). There were 167 patients in cancer Stage II (32.00%), with chemotherapy (175, 34.90%) and surgical treatment (144, 29.70%) as the primary treatment modalities. In terms of lifestyle, 299 patients were smokers (59.70%), and 303 were drinkers (60.50%). Overall, the average age of the gastric cancer patients was 42.40 years (SD = 11.952). Details are presented in Table 1.

**Table 1:** Demographic information of all gastric cancer patients.

**Variables**	**Items**	**Number**	**Proportion**
Gender	Male	299	57.30%
	Female	202	38.70%
Education background	Primary school and below	30	5.70%
	From junior high school to senior high school	284	54.40%
	Bachelor's degree or above	187	35.80%
Monthly income	≤ 1,000¥	43	8.20%
	1,001–3,000¥	112	21.50%
	3,001–5,000¥	196	37.50%
	5,001–8,000¥	130	24.90%
	≥8,001¥	20	3.80%
Place of residence	City	262	50.20%
	Rural	239	45.80%
Cancer staging	Stage I	139	26.60%
	Stage II	167	32.00%
	Stage III	152	29.10%
	Stage IV	43	8.20%
Treatment method	Operative treatment	144	28.70%
	Chemical treatment	175	34.90%
	Radiotherapy	77	15.40%
	Targeted therapy	40	8.00%
	Immunological therapy	65	13.00%
Smoking	Yes	299	59.70%
	No	202	40.30%
Drinking	Yes	303	60.50%
	No	198	39.50%
Age	42.40 ± 11.952		

### 2.2 Research instruments

#### 2.2.1 Death literacy scale

Death literacy was measured using the Chinese version of the Death Literacy Scale, developed by Che et al. (50) and validated for Chinese populations. The scale consists of 29 items across 4 dimensions: knowledge and skills, experiences and practices, value recognition, and social resources and communication. A 5-point Likert scale was used (1 = strongly disagree, 5 = strongly agree), with total scores ranging from 29 to 140; higher scores indicate greater understanding and coping abilities when facing death-related issues. In this study, the Cronbach's α for the Death Literacy Scale was 0.929, demonstrating good internal consistency in the study population. Confirmatory factor analysis showed good model fit, as presented in Table 2.

**Table 2:** Model fit indices for the scales.

**Fit Indices**	**CFI**	**GFI**	**AGFI**	**RMSEA**	**χ^2^/df**	**TLI**
Death literacy	0.919	0.901	0.884	0.044	1.989	0.912
Psychological adaptation	0.998	0.993	0.985	0.015	1.110	0.996
Cognitive reappraisal	1.000	0.994	0.986	0.001	0.973	1.000
Self-management behavior	0.928	0.912	0.899	0.037	1.672	0.923

#### 2.2.2 Psychological adaptation scale

Psychological adaptation was measured using the Brief Adaptation Scale for Stressors (BASE-6), developed by Cruz et al. (51). This scale is a simple tool for assessing individuals' psychological adaptation levels when coping with stressors, comprising 6 items in a single dimension. The items evaluate respondents' emotional stability, sense of life control, problem-solving, and interpersonal interaction abilities over the past two weeks. The scale has been widely applied in Chinese populations, as validated by Sun et al. (52) and Liu and Lin (53), confirming its cultural adaptability. A 5-point Likert scale was used (1 = not at all, 5 = extremely), with total scores ranging from 6 to 30; after reverse scoring, higher scores indicate higher psychological adaptation levels. In this study, the Cronbach's α for the Psychological Adaptation Scale was 0.795, demonstrating good internal consistency in the study population. Confirmatory factor analysis showed good model fit, as presented in Table 2.

#### 2.2.3 Cognitive reappraisal scale

Cognitive reappraisal was assessed using the Cognitive Reappraisal subscale from the Emotion Regulation Questionnaire developed by Gross and John (54). This subscale consists of 6 items in a single dimension, primarily measuring individuals' tendency to regulate emotions by reinterpreting the meaning of situations when facing negative events. The scale was validated for cultural adaptability and reliability in Chinese populations by Chen et al. (55). A 5-point Likert scale was used (1 = strongly disagree, 5 = strongly agree), with total scores ranging from 6 to 30; higher mean scores indicate stronger cognitive reappraisal tendencies. In this study, the Cronbach's α for the Cognitive Reappraisal Scale was 0.777, demonstrating good internal consistency in the study population. Confirmatory factor analysis showed good model fit, as presented in Table 2.

#### 2.2.4 Self-management behavior scale

Self-management behaviors were assessed using the Cancer Patient Self-Management Behavior Assessment Scale developed by Liu et al. (56). Based on social cognitive theory and disease self-management models, the scale comprises 31 items across 4 dimensions: health behavior management, disease cognition and perception, psychological and emotional management, and information acquisition and decision-making participation. Developed for Chinese liver cancer patients, the scale demonstrates good cultural adaptability and reliability. This study used a 5-point Likert scale (1 = never, 5 = always), with total scores ranging from 31 to 155; higher scores indicate higher self-management levels. In this study, the Cronbach's α for the Self-Management Behavior Scale was 0.921, demonstrating good internal consistency in the study population. Confirmatory factor analysis showed good model fit, as presented in Table 2.

### 2.3 Statistical analysis

Data analysis in this study was primarily conducted using SPSS 27.0 and AMOS 29.0 software. The analysis process was divided into four parts: descriptive statistics, correlation analysis, and chain mediation effect analysis. First, descriptive statistics were performed on study variables and demographic characteristics. Continuous data were expressed as mean ± standard deviation (M ± SD), and categorical variables as frequencies and percentages. Skewness (< |3|) and kurtosis (< |8|) within these ranges were considered indicative of normal distribution.

Subsequently, internal consistency (Cronbach's α) was calculated for each scale, and confirmatory factor analysis was used to test structural validity, with evaluation indices including chi-square to degrees of freedom ratio (χ^2^/df), comparative fit index (CFI), goodness-of-fit index (GFI), and root mean square error of approximation (RMSEA); criteria were χ^2^/df < 3.00, CFI and GFI > 0.80, and RMSEA < 0.08 for good model fit. Pearson correlation analysis was employed to explore bivariate correlations between death literacy, psychological adaptation, cognitive reappraisal, and self-management behaviors. Prior to mediation analysis, collinearity diagnostics were conducted on independent and mediating variables, with variance inflation factor (VIF) < 5 indicating no severe multicollinearity.

Finally, a structural equation model was constructed based on stress-coping theory, with death literacy as the independent variable, self-management behaviors as the dependent variable, psychological adaptation and cognitive reappraisal as sequential mediators, and demographic characteristics as controls. Bias-corrected nonparametric percentile bootstrapping (Bootstrap = 5,000 resamples) was used to estimate indirect effects and 95% confidence intervals (CI). If the CI did not include 0, the mediation effect was considered significant. This study utilized the SPSS PROCESS macro (Model 6) to analyze the chain mediating roles of psychological adaptation and cognitive reappraisal.

## 3 Results

### 3.1 Common method bias

Given that all measurement questionnaires in this study utilized 5-point Likert scales, data were sourced from the same location, and responses may be influenced by social desirability, there is a potential for common method bias. Therefore, participant recruitment employed anonymization and data confidentiality measures to mitigate bias.

We conducted Harman's single-factor test by subjecting all variable items to exploratory factor analysis. The results indicated that the first factor accounted for 23.354% of the total variance, which is below the critical threshold of 40%. Thus, this study is free from common method bias.

### 3.2 Descriptive statistics and correlation analysis

Descriptive statistics and correlation analyses were performed on death literacy, psychological adaptation, cognitive reappraisal, and self-management behaviors, as shown in Tables 3, 4. The scores were as follows: death literacy (M = 3.304, SD = 0.623); psychological adaptation (M = 3.429, SD = 0.679); cognitive reappraisal (M = 3.426, SD = 0.657); and self-management behaviors (M = 3.416, SD = 0.597). The core variables all exceeded the critical value (M = 2.5). The skewness ranged from −0.292 to 0.033, and kurtosis ranged from −0.186 to 1.549. According to the approximate normality criteria proposed by Kline (57), the study variables met the assumptions of approximate normality.

**Table 3:** Descriptive statistics of core variables.

**Variables**	**M**	**SD**	**Skewness**	**Kurtosis**
Death literacy	3.304	0.623	−0.200	0.525
Psychological adaptation	3.429	0.679	0.033	−0.184
Cognitive reappraisal	3.426	0.657	−0.121	−0.186
Self-management behavior	3.416	0.597	−0.292	1.549

M represents the mean value, and SD represents the standard deviation.

**Table 4:** Correlation analysis of core variables.

**Variables**	**1**	**2**	**3**	**4**
1. Death literacy	1			
2. Psychological adaptation	0.523^***^	1		
3. Cognitive reappraisal	0.593^***^	0.637^***^	1	
4. Self-management behavior	0.495^***^	0.511^***^	0.561^***^	1

^***^p < 0.001.

As shown in Table 4, death literacy was significantly and positively correlated with psychological adaptation (*r* = 0.523, *p* < 0.001, strong correlation), cognitive reappraisal (*r* = 0.593, *p* < 0.001, strong correlation), and self-management behaviors (*r* = 0.495, *p* < 0.001, moderate correlation). Psychological adaptation was significantly and positively correlated with cognitive reappraisal (*r* = 0.637, *p* < 0.001, strong correlation) and self-management behaviors (*r* = 0.511, *p* < 0.001, strong correlation). Cognitive reappraisal was significantly and positively correlated with self-management behaviors (*r* = 0.561, *p* < 0.001, strong correlation).

### 3.3 Chain mediation analysis

To enhance the accuracy of the study, smoking, alcohol consumption, cancer stage, treatment modality, and gender were included as control variables in the research model. Based on collinearity diagnostics, variance inflation factors < 5 indicated no severe multicollinearity. With death literacy as the independent variable, psychological adaptation and cognitive reappraisal as mediators, and self-management behaviors as the dependent variable, a chain mediation model was constructed. This study employed Process Model 6 to analyze the chain mediating roles of psychological adaptation and cognitive reappraisal (Bootstrap: 5,000 resamples).

The results indicated that death literacy significantly and positively predicted psychological adaptation [β = 0.553, *p* < 0.001, 95% CI = (0.473, 0.633)]; death literacy positively predicted cognitive reappraisal [β = 0.382, *p* < 0.001, 95% CI = (0.305, 0.459)]; and death literacy positively predicted self-management behaviors [β = 0.190, *p* < 0.001, 95% CI = (0.105, 0.276)]. Psychological adaptation significantly and positively predicted cognitive reappraisal [β = 0.445, *p* < 0.001, 95% CI = (0.372, 0.518)]; psychological adaptation positively predicted self-management behaviors [β = 0.195, *p* < 0.001, 95% CI = (0.111, 0.279)]. Cognitive reappraisal significantly and positively predicted self-management behaviors [β = 0.277, *p* < 0.001, 95% CI = (0.188, 0.367)]. Detailed analysis results are presented in Table 5.

**Table 5:** Regression equation model analysis for psychological adaptation and cognitive reappraisal.

**Model**	**Dependent variable**	**Independent variable**	**R**	**R^2^**	**F**	**β**	**t**	**LLCI**	**ULCI**	**VIF**
Model 1	Psychological adaptation	Death literacy	0.565	0.319	38.689^***^	0.553	13.540^***^	0.473	0.633	1.000
Model 2	Cognitive reappraisal	Death literacy	0.711	0.506	72.142^***^	0.382	9.695^***^	0.305	0.459	1.376
		Psychological adaptation				0.445	12.002^***^	0.372	0.518	1.376
Model 3	Self-management behavior	Death literacy	0.622	0.387	38.837^***^	0.190	4.366^***^	0.105	0.276	1.632
		Psychological adaptation				0.195	4.563^***^	0.111	0.279	1.781
		Cognitive reappraisal				0.277	6.079^***^	0.188	0.367	1.997

*R*, multiple correlation coefficient; *R*^2^, coefficient of determination; β, standardized regression coefficient; LLCI/ULCI, lower and upper limits of the 95% confidence interval for the regression coefficient; VIF, variance inflation factor. ^***^p < 0.001.

**Figure 1:**
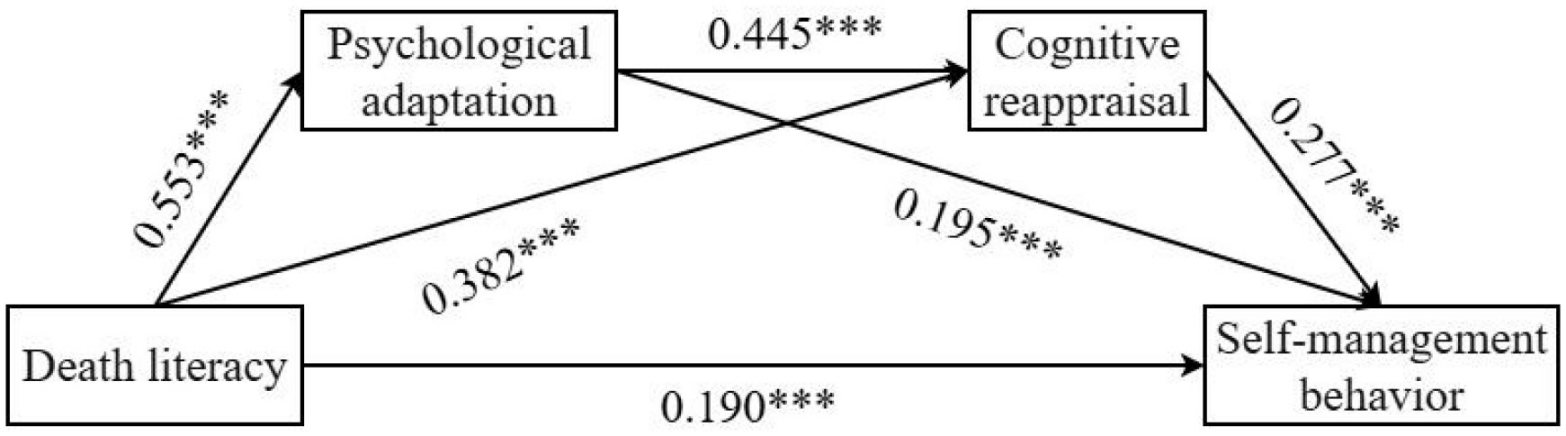
Chain mediation of psychological adaptation and cognitive reappraisal, ***p < 0.001.

The mediation effect analysis, as shown in Table 6 and Figure 1, revealed that the total effect of death literacy on self-management behaviors was 0.472, of which 59.75% was explained by the mediating roles of psychological adaptation and cognitive reappraisal. Further examination showed that the chain mediating path of death literacy → psychological adaptation → cognitive reappraisal → self-management behaviors was significant [β = 0.068, SE = 0.018, 95% CI = (0.031, 0.102)], accounting for 14.40% of the total effect. From the individual mediation paths, death literacy → psychological adaptation → self-management behaviors exhibited a significant mediating effect [β = 0.109, SE = 0.037, 95% CI = (0.036, 0.181)], accounting for 23.09% of the total effect; death literacy → cognitive reappraisal → self-management behaviors exhibited a significant mediating effect [β = 0.106, SE = 0.029, 95% CI = (0.054, 0.170)], accounting for 22.46% of the total effect.

**Table 6:** Decomposition of chain mediation effects.

**Effect**	**β**	**SE**	**LLCI**	**ULCI**	**Effect proportion**	**Supporting hypothesis**
Total effect	0.472	0.038	0.398	0.546	100%	
Direct effect	0.190	0.044	0.105	0.276	40.25%	H1
Total	0.282	0.047	0.191	0.377	59.75%	
Ind1	0.109	0.037	0.036	0.181	23.09%	H2
Ind2	0.106	0.029	0.054	0.170	22.46%	H3
Ind3	0.068	0.018	0.031	0.102	14.40%	H4

Ind1: Death literacy → psychological adaptation → self-management behaviors; Ind2: Death literacy → cognitive reappraisal → self-management behaviors; Ind3: Death literacy → psychological adaptation → cognitive reappraisal → self-management behaviors.

## 4 Discussion

### 4.1 Theoretical implications

This study enriches the social psychological connotations of death literacy from a theoretical perspective by introducing a concept primarily applied in public death education and end-of-life care into the context of psychological and behavioral research on gastric cancer patients. Previous studies have often focused on single-dimensional psychological traits such as death anxiety, death avoidance, or death acceptance (58), whereas this study empirically verifies the positive impact of death literacy on patients' self-management behaviors, revealing that individuals' positive understanding of death at cognitive, emotional, and behavioral levels can translate into specific health management motivations. This finding breaks through the traditional theoretical assumption that death-related variables predominantly exert negative psychological effects, suggesting that death literacy represents not merely a level of cognitive understanding of death issues but a psychological resource for integrating ultimate life meanings, reshaping life goals, and forming behavioral intentions. From a theoretical construction viewpoint, this study confirms that death literacy can serve as an important personality-based psychological foundation for promoting adaptive behavioral changes, further expanding the boundaries of positive death perspectives in behavioral research on cancer patients within health psychology (59), and providing new cognitive coordinates for establishing context-specific death-related health psychological theories in the future.

This study reveals the chain mediating pathway of psychological adaptation and cognitive reappraisal in the process by which death literacy influences self-management behaviors, theoretically deepening the dynamic understanding of psychological regulation mechanisms in cancer patients. Psychological adaptation, as the core process for integrating internal emotions, cognitions, and behavioral responses when facing disease threats, is confirmed to play a key mediating role between death literacy and self-management; this indicates that when patients approach death issues with an open and peaceful attitude, they are more likely to achieve effective life and treatment management through positive psychological integration. Cognitive reappraisal further constitutes a critical psychological mechanism for converting psychological adaptation into health behaviors, reflecting individuals' positive action orientations formed after reinterpreting the meanings of disease events (60). The chain mediation model in this study refines existing cancer psychological regulation theories, demonstrating that positive cognitive processing and emotion regulation strategies serve as bridges connecting meaning construction and behavioral execution, revealing the internal logic of cognition-emotion-behavior linkages, and providing new theoretical fulcrums for understanding how gastric cancer patients rebuild behavioral initiative amid psychological dilemmas.

Grounded in stress-coping theory, this study elucidates the internal psychological mechanisms by which death literacy influences self-management behaviors in gastric cancer patients through psychological adaptation and cognitive reappraisal, expanding the explanatory boundaries of this theory in the field of cancer psychology. Traditional stress-coping models emphasize individuals' cognitive appraisals and coping strategy selections when facing disease threats but primarily focus on stress response alleviation and emotion management (61), with less attention to the regulatory roles of individual meaning construction and existential cognition. By incorporating death literacy into this theoretical framework, this study highlights that individuals' understanding and attitudes toward death not only affect the primary cognitive appraisal process but also profoundly reshape the pathways for reinterpreting the meanings of stressful events. When death is integrated into the life meaning system, individuals are more inclined to adopt positive emotion regulation methods and adaptive coping strategies, thereby promoting the self-maintenance of health behaviors (62). Psychological adaptation and cognitive reappraisal realize a psychological progression mechanism of stress appraisal-emotion regulation-behavior transformation in this process, extending the applicability of stress-coping theory from passive regulation of external pressures to active growth processes based on meaning reconstruction. Consequently, this study proposes a new pathway for psychological stress models in cancer patients: death literacy as a antecedent cognitive resource improves stress appraisals and enhances psychological resilience, facilitating individuals' positive psychological transformations and health behavior maintenance under disease pressures (63). This not only enriches the theoretical connotations of stress-coping theory in chronic disease psychology but also provides new theoretical bases for understanding how cancer patients achieve psychological adaptation and behavioral restoration in high-stress contexts.

### 4.2 Practical implications

This study finds that death literacy has a significant positive promoting effect on self-management behaviors in gastric cancer patients, offering important insights for clinical psychological interventions and health education practices. Traditional oncology interventions often focus on disease control and lifestyle guidance while overlooking patients' psychological construction and meaning understanding of death issues. First, the results demonstrate that death literacy functions as a positive psychological resource that fosters psychological adaptation and self-management behaviors. In practical terms, this highlights the necessity of incorporating structured death education programs into standard oncological care. Such programs should go beyond informational sessions to include guided discussions and reflective exercises, enabling patients to explore their perceptions of mortality and reduce death-related anxiety. For example, clinicians and psychologists can jointly facilitate group-based “life and death dialogue” workshops or narrative therapy sessions during chemotherapy or postoperative rehabilitation. These interventions help patients reframe their understanding of death as part of the life continuum, thereby improving acceptance and internal control. Additionally, a multidisciplinary integration model (MDT-based psychological pathway) is recommended for clinical implementation. Oncologists, psychologists, nurses, and social workers can collaborate to assess individual patients' death literacy and psychological adaptation levels during hospitalization or post-surgery follow-ups. Based on assessment outcomes, individualized psycho-educational plans can be jointly developed and continuously adjusted. Incorporating these modules into digital health platforms or hospital information systems can further enhance accessibility and long-term monitoring of psychological and behavioral outcomes.

The study results indicate that psychological adaptation and cognitive reappraisal play chain mediating roles in the mechanism by which death literacy influences self-management behaviors, providing precise theoretical support for clinical psychological intervention designs. Gastric cancer patients often face complex physiological burdens and psychological conflicts during disease progression, while traditional psychological interventions typically emphasize only disease acceptance or emotional stability, neglecting cognitive processing and meaning reconstruction in coping processes. The findings of this study suggest that clinical psychological support should integrate psychological adaptation training and cognitive reappraisal strategies to help patients achieve positive meaning construction during stress processes. For example, psychologists can employ reappraisal techniques from cognitive behavioral therapy to guide patients in reinterpreting the relationships between disease and life, shifting from uncertainty to growth experiences; simultaneously, psychological adaptation training modules, such as emotion recognition, expressive writing on death-related emotions, and mindfulness relaxation, can be established to promote psychological repair and emotional stability. Through integrated interventions, patients' psychological resilience can be effectively strengthened, enabling them to more capably adjust behaviors proactively to manage symptoms and treatment rhythms, thereby achieving a benign match between psychological mechanisms and health behaviors.

The significant mediating role of psychological adaptation suggests that patients require targeted psychological adaptation training to translate cognitive understanding into emotional stability and behavioral adjustment. This training can consist of structured modules focusing on emotional recognition and regulation, resilience and coping exercises, and goal reorientation and meaning reconstruction. Specific techniques such as cognitive reappraisal exercises, problem-solving therapy, and mindfulness-based stress reduction can be integrated into routine nursing follow-ups or counseling sessions. By strengthening patients' adaptive capacities, these interventions facilitate consistent engagement in diet control, symptom monitoring, and adherence to medical regimens.

The clinical application value of this study lies in revealing the core roles of death literacy and psychological regulation factors in patients' health behaviors, providing theoretical bases for transforming clinical nursing and rehabilitation models. Previous clinical care systems have emphasized physiological treatments and short-term medical adherence while overlooking individuals' internal psychological regulation mechanisms when facing life-and-death issues. This study suggests that healthcare institutions should incorporate death literacy enhancement into hospital humanistic care management systems, implementing human-centered communication principles in diagnostic and therapeutic interactions. For instance, healthcare providers can use open-ended conversation techniques to explore patients' understandings of disease and death, combined with meaning-oriented counseling methods, to reduce anxiety and resistance arising from avoiding death topics. This model transformation not only helps improve doctor-patient trust and treatment adherence but also extends nursing behaviors from biomedical care to psychological and social dimensions, achieving the clinical goal of “holistic care.” By embedding psychological assessment and intervention components into clinical pathways, it can effectively promote self-efficacy and life initiative in gastric cancer patients during disease management processes.

The practical implications of this study also lie in providing promotable theoretical evidence for optimizing psychological health management systems for oncology patients and public health policies. With the increasing trend of cancer as a chronic disease, mere medical interventions can no longer meet patients' comprehensive needs at psychological, social, and value levels. The mechanisms of death literacy, psychological adaptation, and cognitive reappraisal revealed in this study offer scientific bases for developing hierarchical and continuous psychological support systems. Social and health management departments can accordingly establish multilevel psychological service networks in communities and hospitals, combining death education, psychological counseling, and health management courses, while training healthcare personnel in core competencies for death communication and psychological interventions. Simultaneously, this study emphasizes the importance of disease meaning education, which can promote organic connections among “end-of-life care—oncology psychology—health promotion,” facilitating synergistic improvements in patients' quality of life and psychological health. In the future, incorporating death literacy interventions into national standardized pathways for psychological rehabilitation of cancer patients can not only optimize medical resource allocation but also construct culturally adaptive psychological health promotion systems at the macro level, realizing interdisciplinary development from oncology to integrative and social medicine.

### 4.3 Limitations and future research directions

This study employed a cross-sectional design, which can reveal correlations and mediation mechanisms among variables but cannot verify the causal pathways between death literacy, psychological adaptation, cognitive reappraisal, and self-management behaviors in a temporal sequence. Since data were collected at a single time point, individual psychological states and behavioral responses may be influenced by transient emotions, treatment progress, medical environments, and other external factors, thereby limiting the directionality and stability of inferences. Additionally, the study sample was sourced from three tertiary Grade A hospitals in Zhejiang Province, and although it covered patients at different stages and treatment types, the regional characteristics are evident, with the sample generally exhibiting high medical accessibility and relatively high cultural levels, potentially restricting the generalizability of the results. Future research should adopt longitudinal or tracking designs to dynamically monitor the long-term effects of changes in death literacy on self-management behaviors, further validating the temporal and sustained nature of the chain mediation pathway; simultaneously, expanding sample sources to patients from different regions, cultural backgrounds, and medical levels can enhance the model's external validity and cultural adaptability, thereby improving the cross-population promotional value of the results.

This study used self-report questionnaires as the primary data collection method, and although the scales demonstrate high reliability and validity both domestically and internationally, they may still be subject to social desirability bias, self-reporting bias, and recall bias. Some patients may overestimate their psychological adaptation or self-management levels due to social approval motives, thereby interfering with true responses. Furthermore, while the scales for death literacy, psychological adaptation, and cognitive reappraisal have undergone cultural revisions, their specificity for gastric cancer populations requires further validation. Future research recommends integrating multimethodological strategies, such as combining quantitative and qualitative data, through in-depth interviews and focus groups to explore the subjective construction processes of death literacy from patients' life experiences and cultural contexts. Additionally, during data collection, multiple information sources, including observations from family members, caregivers, or clinicians, can be incorporated to form more comprehensive psychological and behavioral profiles. Studies may also adopt physiological or behavioral indicators (e.g., treatment adherence, physical activity monitoring) as objective validations to enhance data ecological validity and reduce methodological biases affecting result interpretations.

Although this study constructed a relatively complete chain mediation model based on stress-coping theory, there remains room for theoretical deepening. First, death literacy in the model primarily serves as a cognitive-level predictor, while its emotional and social dimensions (e.g., death communication experiences, social support systems) may also play key roles in self-management behaviors. Second, this study did not include potential moderating variables such as social support, religious beliefs, cultural orientations, or disease stages, which may exert important interactive effects on model path strengths. Future research can incorporate moderated mediation models or multilevel linear analyses to explore differential effects of various demographic and psychological characteristics on the model's mechanisms. Additionally, variables such as meaning construction and psychological resilience should be integrated into the theoretical framework to further elucidate the functional pathways of death literacy in psychological growth and behavioral reshaping from existential and positive psychology perspectives. By expanding the dimensions of theoretical models, the psychological and behavioral transformation mechanisms from death orientation to life meaning generation in gastric cancer patients can be revealed within broader psychosocial frameworks.

## 5 Conclusion

Based on stress-coping theory, this study systematically reveals the relationships among death literacy, self-management behaviors, and psychological regulation mechanisms in gastric cancer patients, clarifying the chain mediating roles of psychological adaptation and cognitive reappraisal. The results indicate that death literacy is not merely a cognitive ability regarding death knowledge and attitudes but a positive psychological resource that promotes psychological integration and self-regulation. Higher levels of death literacy can enhance psychological adaptation and cognitive reappraisal abilities, helping patients view disease and life more rationally, thereby stimulating positive self-management behaviors and achieving a benign cycle of cognition, emotion, and behavior. This finding not only expands the theoretical application boundaries of death literacy in cancer psychology but also provides new scientific bases for psychological interventions in self-management for gastric cancer patients. From a clinical perspective, the results suggest incorporating death education, psychological adaptation training, and cognitive reappraisal strategies into comprehensive care systems for gastric cancer patients, forming multilevel psychological support pathways to promote patients' long-term self-efficacy and quality of life improvements. Future research can employ longitudinal tracking and cross-cultural comparative methods to further validate the model's stability and generalizability, while exploring the feasibility and sustainability of digital interventions in enhancing patients' death literacy and self-management behaviors.

## Data Availability

The raw data supporting the conclusions of this article will be made available by the authors, without undue reservation.

## References

[1] Van Cutsem E Sagaert X Topal B Haustermans K Prenen H. Gastric cancer. Lancet. (2016) 388:2654–64. doi: 10.1016/S0140-6736(16)30354-3 27156933

[2] Waldum H Fossmark R. Gastritis, gastric polyps and gastric cancer. Int J Mol Sci. (2021) 22:6548. doi: 10.3390/ijms22126548 34207192 PMC8234857

[3] Lin JL Lin JX Lin GT Huang CM Zheng CH Xie JW . Global incidence and mortality trends of gastric cancer and predicted mortality of gastric cancer by 2035. BMC Public Health. (2024) 24:1763. doi: 10.1186/s12889-024-19104-6 38956557 PMC11221210

[4] Wong MCS Huang J Chan PSF Choi P Lao XQ Chan SM . Global Incidence and Mortality of Gastric Cancer, 1980-2018. JAMA Netw Open. (2021) 4:e2118457. doi: 10.1001/jamanetworkopen.2021.18457 34309666 PMC8314143

[5] Wang HM Wang TJ Huang CS Liang SY Yu CH Lin TR . Nutritional status and related factors in patients with gastric cancer after gastrectomy: a cross-sectional study. Nutrients. (2022) 14:2634. doi: 10.3390/nu14132634 35807815 PMC9268084

[6] McShane E Hanna L Zoanetti C Murnane L Baguley B Furness K. The Effect of nutrition impact symptoms on nutrition status after completion of curative-intent treatment for gastric, oesophageal, and pancreatic cancer: a systematic review. Nutrients. (2025) 17:2691. doi: 10.3390/nu17162691 40871719 PMC12389404

[7] Lee JY Hong S. Psychological distress in newly diagnosed patients with gastrointestinal cancer: a scoping review. Asia Pac J Oncol Nurs. (2025) 12:100672. doi: 10.1016/j.apjon.2025.100672 40124661 PMC11930186

[8] Gibson AW Graber JJ. Distinguishing and treating depression, anxiety, adjustment, and post-traumatic stress disorders in brain tumor patients. Ann Palliat Med. (2021) 10:875–92. doi: 10.21037/apm-20-509 32692231

[9] Kavalali Erdogan T Koç Z. Loneliness, death perception, and spiritual well-being in adult oncology patients. Cancer Nurs. (2021) 44:E503–e12. doi: 10.1097/NCC.0000000000000930 33883474

[10] Shahsavar Y Choudhury A. Examining influential factors in newly diagnosed cancer patients and survivors: emphasizing distress, self-care ability, peer support, health perception, daily life activity, and the role of time since diagnosis. PLoS ONE. (2023) 18:e0291064. doi: 10.1371/journal.pone.0291064 37656716 PMC10473484

[11] Gui G Yang D Liu Y Yao Y Xie X Liu R . How family support alleviates death anxiety in breast cancer patients: the mediating role of meaning in life. Front Public Health. (2025) 13:1567485. doi: 10.3389/fpubh.2025.1567485 40236320 PMC11996639

[12] Noonan K Grindrod A Shrestha S Lee S Leonard R Johansson T. Progressing the death literacy index: the development of a revised version (DLI-R) and a short format (DLI-9). Palliat Care Soc Pract. (2024) 18:26323524241274806. doi: 10.1177/26323524241274806 39314871 PMC11418362

[13] Seven A Sari ES Semerci V. Perceptions of good death and death literacy levels of nurses working in palliative care: a cross-sectional study. Int Nurs Rev. (2025) 72:e70036. doi: 10.1111/inr.70036 40401742 PMC12096812

[14] Semerci Çakmak V Seven A Sönmez Sari E. Death anxiety and death literacy among Turkish patients with chronic diseases: a cross-sectional study. BMC Psychiatry. (2025) 25:299. doi: 10.1186/s12888-025-06761-z 40155871 PMC11951526

[15] Rupp SK Stengel A. Influencing factors and effects of treatment on quality of life in patients with gastric cancer-a systematic review. Front Psychiatry. (2021) 12:656929. doi: 10.3389/fpsyt.2021.656929 34276435 PMC8280526

[16] Leonard R Noonan K Horsfall D Kelly M Rosenberg JP Grindrod A . Developing a death literacy index. Death Stud. (2022) 46:2110–22. doi: 10.1080/07481187.2021.1894268 34152939

[17] Yang S Yan C Li J Feng Y Hu H Li Y. The death education needs of patients with advanced cancer: a qualitative research. BMC Palliat Care. (2024) 23:259. doi: 10.1186/s12904-024-01540-1 39516770 PMC11545552

[18] Fitzpatrick PJ. Improving health literacy using the power of digital communications to achieve better health outcomes for patients and practitioners. Front Digit Health. (2023) 5:1264780. doi: 10.3389/fdgth.2023.1264780 38046643 PMC10693297

[19] Bahçecioglu Turan G Türkben Polat H. The effects of illness perception on death anxiety and satisfaction with life in patients with advanced gastrointestinal cancer. Palliat Support Care. (2024) 22:360–6. doi: 10.1017/S1478951523001244 37620999

[20] Bharti A Bharti DA. Impact of death anxiety on psychological well-being and successful aging of older adults with chronic illness. Omega. (2024) 302228241272543. doi: 10.1177/00302228241272543 39107897

[21] Antoni MH Moreno PI Penedo FJ. Stress management interventions to facilitate psychological and physiological adaptation and optimal health outcomes in cancer patients and survivors. Annu Rev Psychol. (2023) 74:423–55. doi: 10.1146/annurev-psych-030122-124119 35961041 PMC10358426

[22] von Blanckenburg P Leppin N Nagelschmidt K Seifart C Rief W. Matters of life and death: an experimental study investigating psychological interventions to encourage the readiness for end-of-life conversations. Psychother Psychosom. (2021) 90:243–54. doi: 10.1159/000511199 33212438

[23] Willis E. Patients' self-efficacy within online health communities: facilitating chronic disease self-management behaviors through peer education. Health Commun. (2016) 31:299–307. doi: 10.1080/10410236.2014.950019 26325224

[24] Mamykina L Smaldone AM Bakken SR. Adopting the sensemaking perspective for chronic disease self-management. J Biomed Inform. (2015) 56:406–17. doi: 10.1016/j.jbi.2015.06.006 26071681 PMC4626451

[25] Lei R Zhang M Gui G Yang D He L. How perceived risk of recurrence strengthens health management awareness in stroke patients: the chain mediating role of risk fear and health literacy. Front Public Health. (2025) 13:1524492. doi: 10.3389/fpubh.2025.1524492 40051512 PMC11882430

[26] Xu A Wang Y Wu X. Effectiveness of e-health based self-management to improve cancer-related fatigue, self-efficacy and quality of life in cancer patients: systematic review and meta-analysis. J Adv Nurs. (2019) 75:3434–47. doi: 10.1111/jan.14197 31566769

[27] Garutti M Noto C Pastò B Cucciniello L Alajmo M Casirati A . Nutritional management of oncological symptoms: a comprehensive review. Nutrients. (2023) 5:5068. doi: 10.3390/nu15245068 38140327 PMC10745914

[28] Kotera Y Green P Sheffield D. Positive psychology for mental wellbeing of UK therapeutic students: relationships with engagement, motivation, resilience and self-compassion. Int J Ment Health Addict. (2022) 20:1611–26. doi: 10.1007/s11469-020-00466-y 33456408 PMC7802612

[29] Gu Y Ma X Xin H Xiang Z Chen Y He C. Navigating life after gastric cancer surgery: a qualitative exploration of the dyadic patient-caregiver perspective on quality of life outcomes. BMC Cancer. (2025) 25:288. doi: 10.1186/s12885-025-13696-x 39966806 PMC11837724

[30] Plotnikoff RC Costigan SA Karunamuni N Lubans DR. Social cognitive theories used to explain physical activity behavior in adolescents: a systematic review and meta-analysis. Prev Med. (2013) 56:245–53. doi: 10.1016/j.ypmed.2013.01.013 23370047

[31] Liu Y Jiang F Zhang M Niu H Cao J Du S . Health literacy and self-management among middle-aged and young hypertensive patients: a parallel mediation effect of illness perception and self-efficacy. Front Psychol. (2024) 15:1349451. doi: 10.3389/fpsyg.2024.1349451 38765827 PMC11099212

[32] Lehti J. Theory of psychological adaptive modes. Med Hypotheses. (2016) 90:66–73. doi: 10.1016/j.mehy.2016.03.003 27063089

[33] Born DO. Psychological adaptation and development under acculturative stress: toward a general model. Soc Sci Med (1967). (1970) 3:529–47. doi: 10.1016/0037-7856(70)90025-9 5483545

[34] Loeffler S Poehlmann K Hornemann B. Finding meaning in suffering? Meaning making and psychological adjustment over the course of a breast cancer disease. Eur J Cancer Care. (2018) 27:e12841. doi: 10.1111/ecc.12841 29575157

[35] Nilsen M Stalsberg R Sand K Haugan G Reidunsdatter RJ. Meaning making for psychological adjustment and quality of life in older long-term breast cancer survivors. Front Psychol. (2021) 12:734198. doi: 10.3389/fpsyg.2021.734198 34650491 PMC8510631

[36] Hu J Wang LL Li Y. Effects of high-quality nursing intervention on negative emotions, postoperative complications and gastrointestinal function in patients with gastric cancer surgery. Am J Transl Res. (2022) 14:1652–62. 35422953 PMC8991151

[37] Christodoulidis G Konstantinos-Eleftherios K Marina-Nektaria K. Double role of depression in gastric cancer: as a causative factor and as consequence. World J Gastroenterol. (2024) 30:1266–9. doi: 10.3748/wjg.v30.i10.1266 38596492 PMC11000075

[38] Akgün Sahin Z Aydin S Kina V. Death literacy, anxiety, and uncertainty in elderly's family caregivers: a cross-sectional study. Omega. (2025) 302228251346910. doi: 10.1177/00302228251346910 40435405

[39] Ting Z Huicai W Kudelati Z Yongkang G Alimu A Xiaotian Z . Exploring the dynamics of self-efficacy, resilience, and self-management on quality of life in type 2 diabetes patients: a moderated mediation approach from a positive psychology perspective. PLoS ONE. (2025) 20:e0317753. doi: 10.1371/journal.pone.0317753 39854536 PMC11759368

[40] Champion VL Skinner CS. The health belief model. In: Health Behavior and Health Education: Theory, Research, and Practice. Vol. 4. San Francisco: Jossey-Bass (2008). p. 45–65.

[41] Cutuli D. Cognitive reappraisal and expressive suppression strategies role in the emotion regulation: an overview on their modulatory effects and neural correlates. Front Syst Neurosci. (2014) 8:175. doi: 10.3389/fnsys.2014.00175 25285072 PMC4168764

[42] Troy AS Shallcross AJ Mauss IB. A person-by-situation approach to emotion regulation: cognitive reappraisal can either help or hurt, depending on the context. Psychol Sci. (2013) 24:2505–14. doi: 10.1177/0956797613496434 24145331

[43] Kubzansky LD Davidson KW Rozanski A. The clinical impact of negative psychological states: expanding the spectrum of risk for coronary artery disease. Psychosom Med. (2005) 67(Suppl 1):S10–4. doi: 10.1097/01.psy.0000164012.88829.41 15953792

[44] Yang D Hu C Zhou Z He L Huang S Wan M . The impact of perceived stigma on appearance anxiety in postoperative rhinoplasty patients: a variable-centered and person-centered perspective. Acta Psychol. (2025) 260:105660. doi: 10.1016/j.actpsy.2025.105660 41086734

[45] Bingley AF McDermott E Thomas C Payne S Seymour JE Clark D. Making sense of dying: a review of narratives written since 1950 by people facing death from cancer and other diseases. Palliat Med. (2006) 20:183–95. doi: 10.1191/0269216306pm1136oa 16764223

[46] Ellsworth PC. Some Implications of Cognitive Appraisal Theories of Emotion. Ann Arbor: University of Michigan Law School (1991).

[47] Atta MHR El-Ashry AM Abd El-Gawad Mousa M. The effects of mindfulness-based techniques on self-rumination, cognitive reappraisal and expressive suppression among patients with major depression: a nursing perspective. J Res Nurs. (2024) 29:366–85. doi: 10.1177/17449871241252011 39291234 PMC11403989

[48] Biggs A Brough P Drummond S. Lazarus and Folkman's psychological stress and coping theory. In: The Handbook of Stress and Health: a Guide to Research and Practice. Hoboken, NJ: Wiley (2017). p. 349–64. doi: 10.1002/9781118993811.ch21

[49] Lazarus RS. Coping theory and research: past, present, and future. Biopsychosoc Sci Med. (1993) 55:234–47. doi: 10.1097/00006842-199305000-00002 8346332

[50] Che SL Li X Zhu M Ng WI. The death literacy index: translation, cultural adaptation, and validation of the Chinese version. Front Public Health. (2023) 11:1140475. doi: 10.3389/fpubh.2023.1140475 37250081 PMC10213892

[51] Cruz RA Peterson AP Fagan C Black W Cooper L. Evaluation of the brief adjustment scale-6 (BASE-6): a measure of general psychological adjustment for measurement-based care. Psychol Serv. (2020) 17:332–42. doi: 10.1037/ser0000366 31169389

[52] Sun X Li BJ Zhang H Zhang G. Social media use for coping with stress and psychological adjustment: a transactional model of stress and coping perspective. Front Psychol. (2023) 14:1140312. doi: 10.3389/fpsyg.2023.1140312 37034939 PMC10075314

[53] Liu Q Lin D. The impact of distance education on the socialization of college students in the Covid-19 era: problems in communication and impact on mental health. BMC Med Educ. (2024) 24:575. doi: 10.1186/s12909-024-05551-7 38789971 PMC11127413

[54] Gross JJ John OP. Individual differences in two emotion regulation processes: implications for affect, relationships, and well-being. J Pers Soc Psychol. (2003) 85:348–62. doi: 10.1037/0022-3514.85.2.348 12916575

[55] Chen S Chi C Luo L Zhu W Chen Y Wang T . Factor structure of the Chinese version of emotion regulation goals scale. Front Psychol. (2024) 15:1392879. doi: 10.3389/fpsyg.2024.1392879 39091708 PMC11292033

[56] Liu B Chen J Zhao X Gui Q Lin Y He B . Development of a self-management behavior assessment scale for liver cancer patients from ethnic minorities. BMC Health Serv Res. (2025) 25:315. doi: 10.1186/s12913-025-12272-0 40001076 PMC11863450

[57] Kline RB. Principles and practice of structural equation modeling. New York, NY: Guilford publications (2023).

[58] Wong SH. Does superstition help? A study of the role of superstitions and death beliefs on death anxiety amongst Chinese undergraduates in Hong Kong. Omega. (2012) 65:55–70. doi: 10.2190/OM.65.1.d 22852421

[59] Aspinwall LG Tedeschi RG. The value of positive psychology for health psychology: progress and pitfalls in examining the relation of positive phenomena to health. Ann Behav Med. (2010) 39:4–15. doi: 10.1007/s12160-009-9153-0 20091429

[60] Shao Y Li WHC Zhou R Cheung AT. The effects of psychological interventions on fostering resilience in family members of pediatric cancer patients: a systematic review and meta-analysis. Cancer Nurs. (2025) 48:490–500. doi: 10.1097/NCC.0000000000001368 38941110

[61] Maqsood A Gul S Noureen N Yaswi A. Dynamics of perceived stress, stress appraisal, and coping strategies in an evolving educational landscape. Behav Sci. (2024) 14:532. doi: 10.3390/bs14070532 39062355 PMC11274181

[62] Clari M Matarese M Ivziku D De Marinis MG. Self-care of people with chronic obstructive pulmonary disease: a meta-synthesis. Patient. (2017) 10:407–27. doi: 10.1007/s40271-017-0218-z 28197788

[63] Ahmadboukani S Fathi D Karami M Bashirgonbadi S Mahmoudpour A Molaei B. Providing a health-promotion behaviors model in elderly: psychological capital, perceived social support, and attitudes toward death with mediating role of cognitive emotion regulation strategies. Health Sci Rep. (2023) 6:e1020. doi: 10.1002/hsr2.1020 36605454 PMC9805290

